# Assessing the relationship between the cerebral metabolic rate of oxygen and the oxidation state of cytochrome-c-oxidase

**DOI:** 10.1117/1.NPh.9.3.035001

**Published:** 2022-07-20

**Authors:** Daniel Milej, Ajay Rajaram, Marianne Suwalski, Laura B. Morrison, Leena N. Shoemaker, Keith St. Lawrence

**Affiliations:** a Lawson Health Research Institute, Imaging Program, London, Ontario, Canada; b Western University, Department of Medical Biophysics, London, Ontario, Canada; c Boston Children’s Hospital, Harvard Medical School, Boston, Massachusetts, United States; d Western University, Department of Kinesiology, London, Ontario, Canada

**Keywords:** near-infrared spectroscopy, diffuse correlation spectroscopy, cerebral blood flow, brain metabolism, cytochrome-c-oxidase

## Abstract

**Significance:**

Hyperspectral near-infrared spectroscopy (hsNIRS) combined with diffuse correlation spectroscopy (DCS) provides a noninvasive approach for monitoring cerebral blood flow (CBF), the cerebral metabolic rate of oxygen (${\mathrm{CMRO}}_{2}$) and the oxidation state of cytochrome-c-oxidase (oxCCO). ${\mathrm{CMRO}}_{2}$ is calculated by combining tissue oxygen saturation ($S_{t}O_{2}$) with CBF, whereas oxCCO can be measured directly by hsNIRS. Although both reflect oxygen metabolism, a direct comparison has yet to be studied.

**Aim:**

We aim to investigate the relationship between ${\mathrm{CMRO}}_{2}$ and oxCCO during periods of restricted oxygen delivery and lower metabolic demand.

**Approach:**

A hybrid hsNIRS/DCS system was used to measure hemodynamic and metabolic responses in piglets exposed to cerebral ischemia and anesthetic-induced reductions in brain activity.

**Results:**

Although a linear relationship was observed between ${\mathrm{CMRO}}_{2}$ and oxCCO during ischemia, both exhibited a nonlinear relationship with respect to CBF. In contrast, linear correlation was sufficient to characterize the relationships between ${\mathrm{CMRO}}_{2}$ and CBF and between the two metabolic markers during reduced metabolic demand.

**Conclusions:**

The observed relationship between ${\mathrm{CMRO}}_{2}$ and oxCCO during periods of restricted oxygen delivery and lower metabolic demand indicates that the two metabolic markers are strongly correlated.

## 1 Introduction

The importance of adequate oxygen metabolism for maintaining cerebral health and function is well recognized considering the high energy demands of the brain—consuming 20% of the body’s total energy budget—and its reliance on blood flow for a constant supply of oxygen and glucose due to a lack of energy stores.^1^ Because of this dependency, inadequate oxygen delivery due to ischemia is a major cause of secondary brain injury in critical care patients recovering from life-threatening neurological emergencies, such as severe traumatic brain injury and subarachnoid hemorrhage.^2^^,^^3^ Independent of inadequate cerebral blood flow (CBF), another cause of secondary brain injury is mitochondrial dysfunction resulting from a hypoxic-ischemic insult. Mitochondrial dysfunction is also a major pathophysiological mechanism of neonatal encephalopathy and the neurologic sequela following cardiac arrest.^4^^,^^5^

Since its initial inception, one of the primary motivations for using near-infrared spectroscopy (NIRS) as a bedside neuromonitoring device has been to assess oxygen metabolism.^6^ The most frequently used parameter is tissue oxygen saturation ($S_{t}O_{2}$), although it is not a direct marker of metabolism because blood oxygenation also depends on CBF.^7^ Based on the Fick principle, the cerebral metabolic rate of oxygen (${\mathrm{CMRO}}_{2}$) can be determined by combining $S_{t}O_{2}$, CBF, and arterial oxygen saturation ($S_{a}O_{2}$) measurements. Initial applications used bolus-tracking methods to measure CBF;^8^^,^^9^ however, the emergence of diffuse correlation spectroscopy (DCS) has enabled continuous ${\mathrm{CMRO}}_{2}$ monitoring.^10^^,^^11^ The combination of NIRS and DCS has been used to measure CBF and ${\mathrm{CMRO}}_{2}$ in clinical studies involving neonatal and adult patient populations.^12^^–^^16^

Due to the unique light-absorbing properties of cytochrome-c-oxidase (CCO), NIRS offers an alternative approach for assessing oxygen metabolism by measuring changes in the oxidation state of CCO (oxCCO).^17^ This metabolic marker is directly related to mitochondrial ATP production and has the advantage of not requiring separate measurements of CBF and $S_{a}O_{2}$, nor does it depend on assuming a fixed arteriovenous blood volume ratio required to convert $S_{t}O_{2}$ to venous oxygen saturation as required to determine ${\mathrm{CMRO}}_{2}$.^18^^,^^19^ Another advantage of the oxCCO is that its concentration is much higher in the brain than in extracerebral tissues; therefore, it is less sensitive to extracerebral layers contamination than the hemoglobin signals. The challenge with monitoring oxCCO is that the *in vivo* concentration of CCO is at least an order of magnitude lower than the hemoglobin concentration.

Broadband or hyperspectral NIRS (hsNIRS) has proven to be the most reliable approach for measuring oxCCO changes accurately,^20^ even in the face of larger oxy- and deoxyhemoglobin signals (${\mathrm{HbO}}_{2}$ and Hb, respectively). The absorption spectrum of the oxCCO is relatively broad (720 to 920 nm) without any strong features, and it overlaps with the hemoglobins’ spectra. Consequently, the multiwavelength approach improves the reliability of the measured oxCCO changes. Clinical studies have shown the ability of hsNIRS to detect oxCCO changes associated with neonatal hypoxic-ischemic encephalopathy, preterm birth, and cardiac surgery.^21^^–^^25^

A disadvantage with measuring oxCCO is the lack of a suitable commercial monitor given the requirement of hyperspectral measurements to minimize crosstalk between chromophores. In contrast, a number of NIRS/DCS devices, some of which are available commercially, have been developed to quantify CBF and $S_{t}O_{2}$.^26^^,^^27^ Considering that ${\mathrm{CMRO}}_{2}$ and the redox state of CCO both reflect cellular oxygen metabolism, it is reasonable to expect that these two parameters should be correlated, provided the electron transport chain has a ready supply of electrons from substrates (primarily NADH, nicotinamide adenine dinucleotide (NAD) + hydrogen (H)), and there is no change in CCO concentration.^28^ To test this hypothesis, this study consisted of two experiments aimed at altering cerebral oxygen metabolism. Both sets of experiments were conducted in newborn piglets to avoid signal contamination from extracerebral tissues. In the first set, carotid occlusion was used to restrict oxygen delivery to the brain, which causes a corresponding decrease in metabolism.^29^ In the second set, cerebral metabolic demand was reduced without impeding CBF by altering the anesthetics administered to the animals.^8^ Experiments were performed using a hybrid hsNIRS/DCS system capable of collecting CBF, ${\mathrm{HbO}}_{2}$, Hb, and oxCCO data simultaneously. In addition, absolute baseline CBF was measured by a bolus-tracking method to convert DCS measurements into physiological units of blood flow.^30^

## 2 Methods

### 2.1 Instrumentation

All data were collected using an in-house built hsNIRS/DCS system that incorporated a multiplexing shutter system to cycle between data acquisition from the two subsystems.^23^^,^^24^ The NIRS light source was a 20-W halogen bulb (Hl-2000-HP, Ocean Optics) that was filtered from 600 to 1000 nm and coupled into a custom optical fiber bundle (active diameter 2.4 mm, fiber core 30  μM, NA=0.55, Loptek, Germany) to direct the light to the head. Reflected broadband light from the interrogated tissue was collected at a source–detector distance of 3 cm by three fiber bundles (bundle active diameter 2 mm, fiber core diameter 30  μm, NA=0.55, Loptek, Germany) that were linearly aligned at the entrance of the spectrometer (iDus 420, $\lambda_{\text{Bandwidth}}=548$ to 1085 nm, $\lambda_{\text{Resolution}}=1.65\;\mathrm{nm}$; Andor, Oxford Instruments, Canada).

For the DCS module, light from a continuous laser with a long coherence length (>10  m) emitting at λ=785  nm (DL785-100s, CrystaLaser) was coupled into a single fiber (fiber core diameter 400  μm, NA=0.22, Loptek, Germany). On the detection side, light was collected at a source–detector distance of 2 cm by single-mode fibers (core diameter 8  μm, NA=0.12, Loptek, Berlin, Germany) coupled to a four-channel single-photon counting module (SPCM-AQR-15-FC, Excelitas Technologies, Canada). Each counting module generated TTL pulses sent to an edge-detecting photon counter on a PCIe6612 counter/timer data acquisition board (National Instrument).^27^^,^^31^ Photon counts were recorded and processed using in-house developed software written in LabVIEW (2017 SP1, National Instruments) and MATLAB (2016b, MathWorks). Intensity autocorrelation curves were generated for each detector at 40 delay times (τ) ranging from 1 to 40  μs.^32^

### 2.2 Animal Preparation

Animal experiments were conducted in accordance with the guidelines of the Canadian Council of Animal Care (CCAC) and approved by the Animal Care Committee at Western University. Young piglets (age ranged from 12 to 216 h) were initially anesthetized with 5% isoflurane, which was reduced to 3% for surgical procedures and maintained at 2% to 3% during the experiment. Piglets were tracheotomized and mechanically ventilated on an oxygen-medical air mixture. Incisions were made lateral to the trachea, and vascular occluders (In Vivo Metric, California were placed around the carotid arteries posterior to the clavicle. Catheters were inserted into both ear veins for injections and into a femoral artery to collect arterial blood samples for gas and glucose analyses and for monitoring arterial blood pressure. Vital signs [heart rate (HR), $S_{a}O_{2}$, end-tidal ${\mathrm{CO}}_{2}$ tension, respiratory rate, blood pressure, and temperature] were monitored continuously (SurgiVet, Smiths Medical, Minnesota). Piglets were placed in the prone position with the hsNIRS and DCS probes secured to the top of the head using an in-house three-dimensional-printed probe holder.

### 2.3 Experimental Procedure

After a stabilization period of ∼30  min following surgery, a bolus of propofol (6.4  mg/kg) was administered intravenously to reduce cerebral energy metabolism.^33^ After a second delay of ∼1  h to allow propofol effects to wear off, the carotid occluders were inflated to reduce global CBF and released after 10 min to restore normal blood flow. The hybrid system was used to collect broadband and DCS data throughout the two procedures continuously. The shutter system cycled between the hsNIRS and DCS systems in 3-s intervals with an additional 0.5-s delay added between intervals to account for shutter transition times and avoid any cross contamination between the two systems. For both systems, data were acquired at 4 Hz during each 3-s interval, which were subsequently averaged to generate a single measurement every 7 s. The final step was to measure absolute CBF by dynamic contrast-enhanced NIRS. The protocol consisted of an intravenous bolus injection of indocyanine green (ICG, 0.1  mg/kg). The rapid passage of the dye through the brain was recorded by continuous hsNIRS acquisition at a temporal resolution of 250 ms. The corresponding time-varying arterial ICG concentration, required for flow quantification, was measured using a dye densitometer attached to a front paw (DDG 2001, Nihon Kohden, Japan).

### 2.4 Data Analysis

#### 2.4.1 Hyperspectral NIRS analysis

Details of the hsNIRS data analysis have been described previously.^29^ For each animal, a reference spectrum (${\text{reference}}_{\lambda }$) and a dark count spectrum (${\text{dark}}_{\lambda }$) were acquired at the beginning of each experiment. A baseline reflectance spectrum was determined from spectra (${\text{data}}_{\lambda }$) collected prior to propofol infusion using the following:

$$R(\lambda )=\log_{10}(\frac{{\text{data}}_{\lambda }-{\text{dark}}_{\lambda }}{{\text{reference}}_{\lambda }-{\text{dark}}_{\lambda }}).$$ (1)

The first and second derivatives of R(λ) were fit with the solution to the diffusion approximation for a semi-infinite homogeneous medium^34^ to generate estimates of the tissue water fraction, ${\mathrm{HbO}}_{2}$ and Hb concentrations, and two scattering parameters [wavelength-dependent power and the reduced scattering coefficient (${\mu_{s}}^{\prime }$) at 800 nm].^24^ Fitting was performed using a constrained minimization algorithm based on the MATLAB function fminsearchbnd. The estimates of ${\mathrm{HbO}}_{2}$ and Hb concentrations were used to calculate baseline tissue oxygen saturation ($S_{t}O_{2}^{b}$).

A modified Beer–Lambert law based on the UCLn algorithm was used to calculate subsequent changes in Hb, ${\mathrm{HbO}}_{2}$, and oxCCO concentrations.^17^ For this analysis, the differential pathlengths for each animal were calculated by fitting the second derivative of the average baseline R(λ) to the second derivative of the water absorption spectrum^35^ and correcting for the wavelength dependence of the pathlength.^36^ The UCLn algorithm was performed twice. First, to quantify changes in oxCCO concentration (ΔoxCCO) from attenuation changes between 770 and 906 nm.^17^ This wavelength range is optimized for oxCCO as it avoids the large deoxyhemoglobin absorption feature at λ=760  nm.^37^ Next, the analysis was repeated for the wavelength range between 680 and 850 nm to determine changes in Hb and ${\mathrm{HbO}}_{2}$ concentrations.^38^ It was previously shown that this range provides a good assessment of tissue saturation because absorption changes are dominated by oxy and deoxyhemoglobin.^39^ Tissue oxygen saturation at each time point was determined by combining the relative changes derived from the UCLn algorithm with the absolute baseline value obtained by derivative spectroscopy. The resulting times series of ΔHb, $\Delta {\mathrm{HbO}}_{2}$, and ΔoxCCO were smoothed with a zero-phase digital filter (filtfilt, MATLAB, 2016b, MathWorks).

#### 2.4.2 DCS analysis

Normalized intensity autocorrelations functions were converted to electric field autocorrelation data using the Siegert relation.^40^ Each autocorrelation function was subsequently fit with the solution to the diffusion approximation for a semi-infinite homogenous medium. The fitting incorporated coherence factor (β) dynamic $\mu_{a}$ measurements obtained by hsNIRS and assuming ${\mu_{s}}^{\prime }=8\;{\mathrm{cm}}^{-1}$.^41^ An assumed value of ${\mu_{s}}^{\prime }$ was used instead of the estimate from the analysis of the hsNIRS data, as differential spectral analysis has been shown to underestimate ${\mu_{s}}^{\prime }$.^34^ The fitting variable was a blood flow index (BFi) based on modelling tissue perfusion as a pseudo-Brownian motion.^10^ Fitting was performed across all correlation times from 1 to 40  μs. The resulting BFi time courses were smoothed with the same filter (zero-phase digital filtering, filtfilt, MATLAB, 2016b, MathWorks) as the hsNIRS data.

#### 2.4.3 Dynamic contrast-enhanced NIRS

Baseline CBF was quantified by relating the time-varying arterial concentration of ICG, $C_{a}(t)$, to the corresponding brain concentration curve, $C_{b}(t)$:

$$C_{b}(t)=CBF\cdot R(t)*C_{a}(t),$$ (2)

where R(t) is the impulse residue function and ∗ refers to the convolution operator. The product CBF · R(t) was extracted by deconvolution,^42^ from which CBF was determined by the initial value, for R(t) by definition equals 1. The same fitting algorithm used to determine the baseline optical properties was used to determine Q(t) with ICG concentration as the only fitting parameter.

#### 2.4.4 Calculating the cerebral metabolic rate of oxygen

The final step was to calculate the ${\mathrm{CMRO}}_{2}$ (ml $O_{2}/100\;g/\min$), which was based on the standard mass balance equation relating ${\mathrm{CMRO}}_{2}$ to CBF and $S_{t}O_{2}$:^32^^,^^43^

$${\mathrm{CMRO}}_{2}(t)=\mathrm{CBF}(t)\cdot \frac{K\cdot \mathrm{tHb}}{f_{v}}\cdot (S_{a}O_{2}-S_{t}O_{2}(t)),$$ (3)

where K is the oxygen-carrying capacity of hemoglobin (1.39 ml of $O_{2}$ per g Hb);^44^ tHb is the total hemoglobin concentration, which was determined from the baseline arterial blood sample; and $f_{v}$ is the venous volume fraction, which was set to 0.75.^32^^,^^43^ The CBF time series CBF(t) in physiological units of blood flow (i.e., ml/100  g/min) was generated by scaling BFi data by baseline CBF measured by contrast-enhanced NIRS.

### 2.5 Statistical Analysis

All data are presented as mean ± standard error, and statistical significance was defined as p<0.05.

The relationships between changes in each metabolic parameter and CBF and between the two metabolic parameters were assessed using a least-squares algorithm to fit a power-law relationship:

$$\Delta Y={\left(k\cdot \Delta X\right)}^{\alpha },$$ (4)

where k is a scaling factor, α is the power exponent, ΔY was either the change in ${\mathrm{CMRO}}_{2}$ ($\Delta {\mathrm{CMRO}}_{2}$) or ΔoxCCO, and ΔX was either the change in CBF (ΔCBF) or $\Delta {\mathrm{CMRO}}_{2}$. For this analysis, time series were converted into bins that had a width of 1  ml/100  g/min when ΔCBF was the independent variable and $0.01\;\mathrm{ml}\;O_{2}/100\;g/\min$ when $\Delta {\mathrm{CMRO}}_{2}$ was the independent variable. A paired t-test was used to determine if the mean value of α for each test was statistically different from one. Linear regression was used in cases in which the difference did not reach significance.

## 3 Results

Data were acquired from six piglets. Relevant baseline physiological parameters are provided in Table 1. Each experiment, including both procedures, took on average 4.72±0.49  h to complete. Average baseline CBF and ${\mathrm{CMRO}}_{2}$ measured by dynamic contrast-enhanced NIRS were 53.3±5.7  ml/100  g/min and $1.7\pm 0.9\;{\text{ml}\;\text{O}}_{2}/100\;g/\min$, respectively.

**Table 1:** Physiological parameters.

Parameter	Piglet no.					
	1	2	3	4	5	6
Sex	Female	Male	Male	Female	Female	Female
Age (h)	216	12	60	48	24	12
Weight (kg)	4.1	1.9	2.7	2.5	1.7	2.0
Blood pressure (mmHg)	46	40	38	44	45	34
HR ($\min^{-1}$)	166	146	138	169	163	129
${\mathrm{SaO}}_{2}$ (%)	95	99	98	98	98	99
tHb (g/dl)	4.5	6.1	8.0	7.2	6.3	6.7
Temperature (°C)	37.7	38.6	37.9	38.5	38.5	37.9
$P_{a}{\mathrm{CO}}_{2}$ (mmHg)	35.3	37.7	40.0	36.6	37.7	35.9
$P_{a}O_{2}$ (mmHg)	265	263	260	186	260	324
Respiratory rate ($\min^{-1}$)	42	55	51	51	55	45
Glucose (mM)	10.5	7.7	5.2	7.1	5.5	6
${\mathrm{CBF}}_{0}$ (ml/100 g/min)	51.5	57.0	59.8	57.7	46.2	44.9
${\mathrm{CMRO}}_{2,0}$ ($\mathrm{ml}{\;O}_{2}/100\;g/\min$)	1.29	3.19	1.73	1.44	0.55	0.76

${\mathrm{SaO}}_{2}$, arterial oxygen saturation; ${\mathrm{PaCO}}_{2}$, arterial partial pressure of carbon dioxide; ${\mathrm{PaO}}_{2}$, arterial partial pressure of oxygen; tHb, total hemoglobin concentration; ${\mathrm{CBF}}_{0}$, baseline cerebral blood flow; ${\mathrm{CMRO}}_{2,0}$, baseline cerebral metabolic rate of oxygen.

**Fig. 1:**
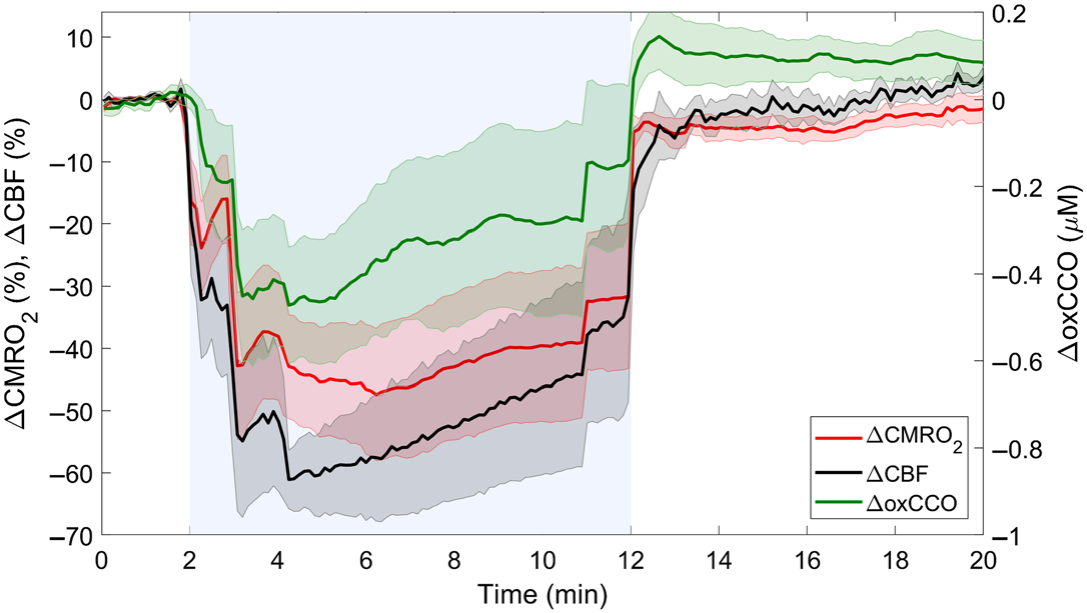
Average temporal changes in CBF, ${\mathrm{CMRO}}_{2}$, and oxCCO in response to carotid occlusion, which is indicated by the blue region between 2 and 12 min. Time courses were averaged across animals, and shading surrounding each line represents the standard error of the mean.

Figure 1 presents average changes in CBF, ${\mathrm{CMRO}}_{2}$, and oxCCO during carotid occlusion. All three parameters exhibited rapid changes at the beginning and end of the occlusion period. The biphasic responses observed at these transition periods were a consequence of sequential inflating/releasing of the occluders. Complete carotid occlusion resulted in a maximum CBF reduction of 38.8±7.7  ml/100  g/min or 69.5±9.2% relative to baseline. The corresponding ${\mathrm{CMRO}}_{2}$ nadir was $0.9\pm 0.7\;\text{ml}\;O_{2}/100\;g/\min$ or 47.7±18.5%, and the maximum reduction in oxCCO was 0.7±0.2  μM.

**Fig. 2:**
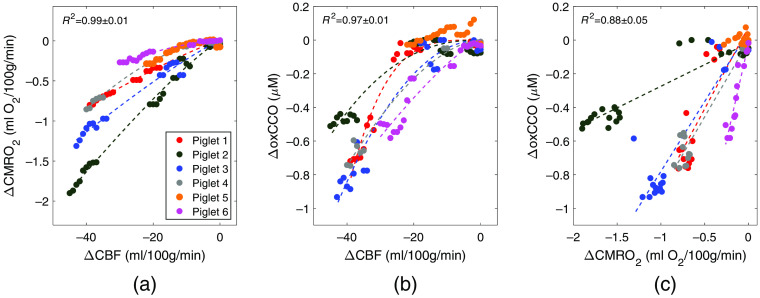
Correlation plots showing the relationship between changes in the two metabolic parameters ($\Delta {\mathrm{CMRO}}_{2}$ and ΔoxCCO) and corresponding changes in CBF (ΔCBF) in response to carotid occlusion: (a) $\Delta {\mathrm{CMRO}}_{2}$ versus ΔCBF and (b) ΔoxCCO versus ΔCBF. Plot (c) presents the relationship between $\Delta {\mathrm{CMRO}}_{2}$ and ΔoxCCO. Each color indicates data from an individual animal. Dashed lines represent the best-fit lines of the power law in (a) and (b) and linear regression in (c).

Figure 2 presents the results of the regression analysis used to determine the relationships among the three parameters during carotid occlusion: (a) $\Delta {\mathrm{CMRO}}_{2}$ and ΔCBF, (b) ΔoxCCO and ΔCBF, and (c) ΔoxCCO and $\Delta {\mathrm{CMRO}}_{2}$. The analysis was conducted for each animal separately, and all changes are relative to preocclusion values. The results from one animal (piglet 5, orange symbols in Fig. 2) were not included in the regression analysis as the reduction in CBF was modest (ΔCBF=37%) compared with the other animals (ΔCBF=70±7%) with no appreciable change in oxCCO, which was likely due to insufficient occlusion. To investigate the potential influence of inter-subject variability in the physiological parameters (Table 1) on the reconstructed CBF changes, linear regression was conducted between each physiological parameter and ΔCBF, and no statistically significant relationship was found for any of the parameters. Based on the remaining five animals, the average exponent of the power law (α) was 1.39±0.31 for $\Delta {\mathrm{CMRO}}_{2}$ versus ΔCBF and 2.23±0.92 for ΔoxCCO versus ΔCBF. These values were significantly greater than one but not significantly different from each other. In contrast, the mean value of α characterizing the relationship between ΔoxCCO and $\Delta {\mathrm{CMRO}}_{2}$ was not statistically different from one, and therefore linear regression was used to characterize this relationship. Results of the regression analysis for each animal, including the coefficient of determination, are presented in Table 2.

**Table 2:** Best-fit estimates of the power law exponent (α) from individual animals. Results from linear regression for $\Delta \mathrm{oxCCO}/\Delta {\mathrm{CMRO}}_{2}$ are also presented.

Animal no.	$\Delta {\mathrm{CMRO}}_{2}/\Delta \mathrm{CBF}$		ΔoxCCO/ΔCBF		$\Delta \mathrm{oxCCO}/\Delta {\mathrm{CMRO}}_{2}$		
	α	$R^{2}$	A	$R^{2}$	Slope	Intercept	$R^{2}$
1	1.18	1.00	3.71	0.98	0.97	0.08	0.81
2	1.17	1.00	2.37	0.96	0.26	−0.02	0.88
3	1.15	0.99	2.07	0.97	0.83	0.05	0.88
4	1.79	1.00	1.71	0.99	0.89	−0.01	0.95
6	1.67	0.99	1.27	0.96	2.28	−0.03	0.88

**Fig. 3:**
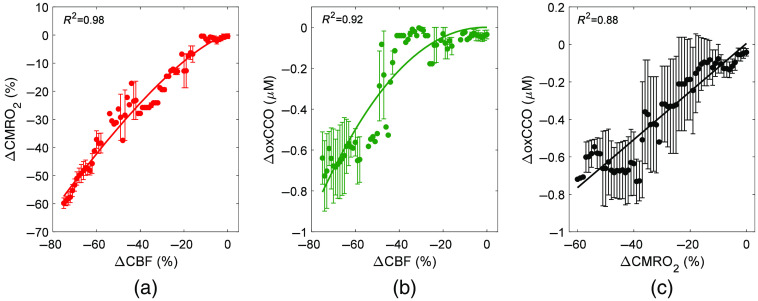
Relationship between relative metabolic and blood flow changes in response to carotid occlusion: (a) $\Delta {\mathrm{CMRO}}_{2}$ versus ΔCBF, (b) ΔoxCCO versus ΔCBF, and (c) $\Delta {\mathrm{CMRO}}_{2}$ versus ΔoxCCO. Data were averaged across five animals, and the solid lines represent the best fit of either the power law in (a) and (b) or linear regression in (c). Error bars represent the standard deviation across animals.

Figure 3 shows the relationships between the three parameters during carotid occlusion averaged across animals. Relative changes are presented to remove the variability observed between animals in Fig. 2. Best-fit estimates of α were 1.37 for $\Delta {\mathrm{CMRO}}_{2}$ versus ΔCBF and 2.14 for ΔoxCCO versus ΔCBF. Linear regression revealed a strong correlation between ΔoxCCO and $\Delta {\mathrm{CMRO}}_{2}$ with a slope of 0.013  μM ΔoxCCO per % $\Delta {\mathrm{CMRO}}_{2}$ and an intercept of ΔoxCCO=0.028  μM.

**Fig. 4:**
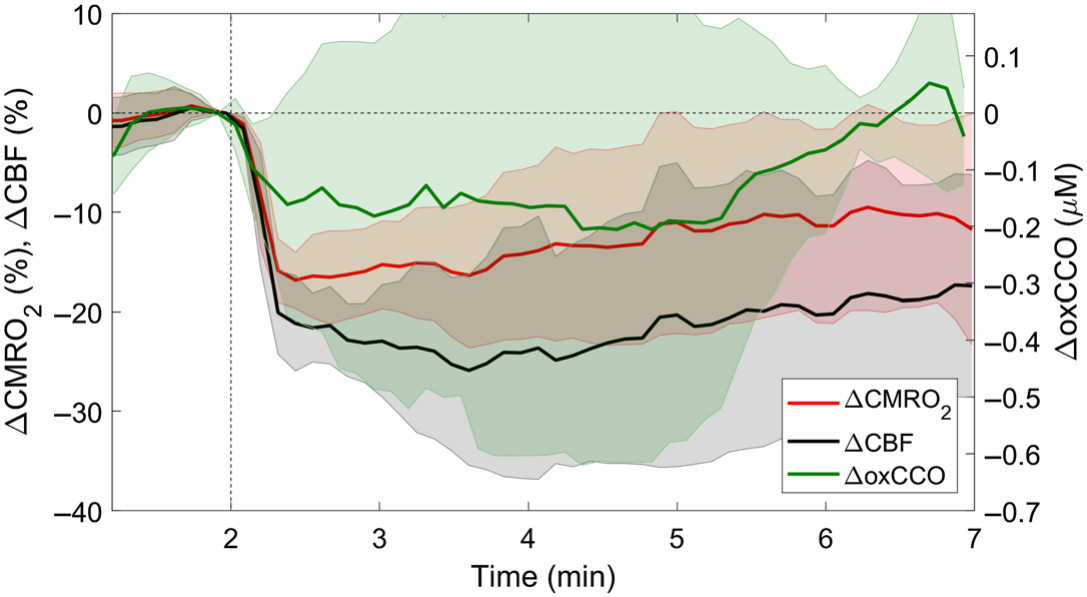
Average changes in CBF, ${\mathrm{CMRO}}_{2}$, and oxCCO in response to a bolus injection of propofol (6.4  mg/kg) administered at the 2-min mark (indicated by the vertical dashed line). Each time course was averaged across six animals, and the shading surrounding each line represents the standard error of the mean.

Figure 4 presents average changes in CBF, ${\mathrm{CMRO}}_{2}$, and oxCCO in response to propofol injection. The maximum CBF reduction was 16.7±5.2  ml/100  g/min (29.5±7.2%), with a corresponding decrease in ${\mathrm{CMRO}}_{2}$ of $0.5\pm 0.3\;\text{ml}\;O_{2}/100\;g/\min$ (20.6±4.5%) and oxCCO of 0.27±0.2  μM.

**Fig. 5:**
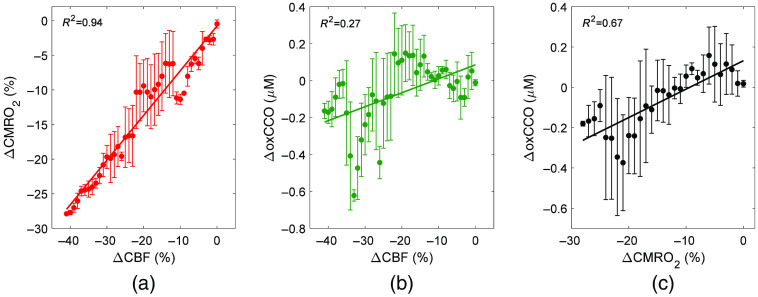
Correlation plots of the relationship between (a) $\Delta {\mathrm{CMRO}}_{2}$ and ΔCBF, (b) ΔoxCCO and ΔCBF, and (c) $\Delta {\mathrm{CMRO}}_{2}$ and ΔoxCCO in response to propofol injection. The solid lines represent the results of linear regression: (a) $R^{2}=0.93$, slope=0.65 $\left(\%\Delta {\mathrm{CMRO}}_{2})/(\%\Delta \mathrm{CBF}\right)$, intercept=−0.49 (% ΔCBF); (b) $R^{2}=0.28$, slope=0.007  μM/(%ΔCBF), intercept=0.086  μM; and (c) $R^{2}=0.72$, $\text{slope}=0.014\;\mu M/(\%\Delta {\mathrm{CMRO}}_{2})$, intercept=0.013  μM. Error bars represent the standard deviation across animals.

Compared with carotid occlusion, the decreases in CBF, ${\mathrm{CMRO}}_{2}$, and oxCCO following propofol injection were relatively small. Consequently, linear regression analysis was used to characterize the relationship between the metabolic parameters and CBF instead of the power law (Fig. 5). The analysis revealed a strong correlation between changes in CBF and ${\mathrm{CMRO}}_{2}$; however, only a weak-to-moderate relationship was observed between ΔCBF and ΔoxCCO. Finally, a moderate-to-strong linear relationship was found between ΔoxCCO and $\Delta {\mathrm{CMRO}}_{2}$.

## 4 Discussion

The motivation for this study was to investigate the relationship between the two metabolic markers that can be assessed by NIRS: the cerebral metabolic rate of oxygen and the oxidation state of cytochrome-c-oxidase. The main finding was that $\Delta {\mathrm{CMRO}}_{2}$ and ΔoxCCO remained coupled when oxygen delivery to the brain was restricted and when the cerebral metabolic demand was lowered.

The mean CBF time course shown in Fig. 1 illustrates the expected decrease in global cerebral perfusion caused by carotid occlusion. On average, CBF decreased by ∼70%, corresponding to an average value of 35.9±9.1  ml/100  g/min at its nadir. This value is within the range reported for the ischemic threshold,^45^ although brain injury from this type of whole-brain insult typically requires durations longer than 10 min and the combination of hypoxia and ischemia.^46^ Nevertheless, the correlation plots in Figs. 2 and 3 demonstrate that the impact on oxygen delivery was significant enough to cause sizable reductions in ${\mathrm{CMRO}}_{2}$ and oxCCO. Power law analysis revealed that both decreases in ${\mathrm{CMRO}}_{2}$ and oxCCO exhibited a nonlinear relationship with respect to concurrent decreases in CBF. Similar findings have been previously reported for ${\mathrm{CMRO}}_{2}$ during middle cerebral artery occlusion in pigs and for oxCCO in a piglet model of hypoxia-ischemia and in patients during cardiac surgery.^23^^,^^29^^,^^47^ The dampened metabolic responses reflect the compensatory increase in oxygen extraction that occurs in an effort to match energy demands.^48^ In contrast, a strong linear correlation between decreases in ${\mathrm{CMRO}}_{2}$ and oxCCO was found ($R^{2}=0.88$), indicating that impeding oxygen delivery had a similar effect on both markers. The results presented in Fig. 2 showed a large variation in the dynamic relationship between the two metabolic markers. Two extrema can be observed (animals 2 and 6) in which the amplitudes of the responses were opposite for the two metabolic markers. The ${\mathrm{CMRO}}_{2}$ values given in Table 1 show that these two animals also had the lowest (#6) and highest (#2) baseline values. To investigate the potential influence of the baseline oxygen consumption on the relationship between the two metabolic markers, we conducted a linear regression between ${\mathrm{CMRO}}_{2,0}$ and the $\Delta \mathrm{oxCCO}/\Delta {\mathrm{CMRO}}_{2}$ slopes given in Table 2. A moderate-to-strong linear relationship was found ($R^{2}=0.69$), but it did not reach statistical significance (p=0.08). These results suggest that the metabolic response in the brain to reduced oxygen delivery does depend on the initial baseline metabolic rate.

The reduction in brain activity caused by injecting the anesthetic propofol resulted in a different relationship between cerebral perfusion and metabolism compared with that observed during carotid occlusion. In this case, the lower metabolic demand was matched by a similar reduction in CBF, as evident by the strong linear correlation between CBF and ${\mathrm{CMRO}}_{2}$ (Fig. 5; $R^{2}=0.93$). This finding of proportional decreases in CBF and ${\mathrm{CMRO}}_{2}$ following propofol infusion agrees with previous studies.^33^^,^^49^ Overall, the effects of propofol were small compared with carotid occlusion, with roughly 50% smaller reductions in CBF, ${\mathrm{CMRO}}_{2}$, and oxCCO. Most likely, the background isoflurane anesthesia tempered the effects of propofol. Only a weak correlation was found between ΔoxCCO and ΔCBF ($R^{2}=0.28$) due to the considerable variability observed in the ΔoxCCO data (Fig. 4) as the small magnitude of the changes was close to the precision limits of the measurements. It is possible that the direct effects of propofol on oxidative substrate levels could have counteracted the reduction in oxCCO^50^^,^^51^ caused by lowering cerebral energy demands. However, the decreases in oxCCO for the two sets of experiments were similar for the same change in CBF. Despite the weak correlation between ΔoxCCO and ΔCBF, a moderate-to-strong correlation ($R^{2}=0.72$) was found between $\Delta {\mathrm{CMRO}}_{2}$ and ΔoxCCO. In agreement with these experimental results, a model designed to simulate the regulation of brain hemodynamics and metabolism predicts a linear relationship between ${\mathrm{CMRO}}_{2}$ and oxCCO during modest changes in energy demands (i.e., ±20% changes in ${\mathrm{CMRO}}_{2}$).^28^

The results of this study should not be interpreted as suggesting that ${\mathrm{CMRO}}_{2}$ and oxCCO are always coupled. Indeed, previous studies have illustrated conditions in which changes in oxCCO occur with no measurable difference in ${\mathrm{CMRO}}_{2}$. The treatment of patent ductus arteriosus in preterm infants by the cyclooxygenase inhibitor indomethacin reduces oxCCO^52^ but does not affect ${\mathrm{CMRO}}_{2}$ despite the vasodilatory effects of the drug.^14^^,^^53^ Likewise, moderate hypercapnia increases oxCCO,^54^ along with CBF, but is generally considered not to alter ${\mathrm{CMRO}}_{2}$.^32^^,^^55^ These discrepancies indicate that some conditions can lead to fluctuations in intracellular oxygen tension that are not sufficient to affect the mitochondrial respiratory rate. A more complete understanding of the intricate relationship between ${\mathrm{CMRO}}_{2}$ and oxCCO should account for other variables that can influence the oxCCO signal, including the availability of respiratory electrons and the rate of ATP utilization.^17^ Imaging of glucose metabolism could be used to gauge substrate supply and phosphorus magnetic resonance spectroscopy to assess the rate of ATP hydrolysis.^56^^,^^57^

A limitation of this study was the small sample size. Five successful experiments were sufficient to show that the relationship between the two metabolic parameters and CBF during carotid occlusion were better characterized by the power law than linear regression. However, the study did not have sufficient power to determine if the two responses were different as suggested by their exponents (α=1.39±0.31 for $\Delta {\mathrm{CMRO}}_{2}/\Delta \mathrm{CBF}$ and 2.23±0.92 for ΔoxCCO/ΔCBF). Based on these average and standard deviations values, power analysis indicated that an additional five data sets would be required to assess statistical difference between the metabolic markers. The higher exponent for ΔoxCCO/ΔCBF agrees with the concept that low oxygen tension is required to reduce CCO.^58^ In terms of $\Delta {\mathrm{CMRO}}_{2}/\Delta \mathrm{CBF}$, a potential confounding factor could be applying the Fick principle [i.e., Eq. (3)], which assumes a steady-state, to dynamic CBF and $S_{t}O_{2}$ data. Furthermore, possible changes in the assumed venous fraction ($f_{v}$), which was fixed to 0.75, would also affect the accuracy of the $\Delta {\mathrm{CMRO}}_{2}$ measurements.^19^

## 5 Conclusions

In summary, combining hsNIRS with DCS provided a means of assessing the relationship between ${\mathrm{CMRO}}_{2}$ and oxCCO. Moderate-to-strong linear correlations were found between the two metabolic parameters in response to reductions in global CBF and brain activity. These results indicate that, under these conditions, ${\mathrm{CMRO}}_{2}$ and oxCCO provided similar information regarding cerebral energy metabolism. More studies are required to determine if this agreement would extend to clinically relevant conditions such as perinatal hypoxia-ischemia, considering mitochondrial dysfunction is a major contributor to delayed brain injury.^19^^,^^21^ Given the differences in technologies required to monitor ${\mathrm{CMRO}}_{2}$ and oxCCO, it would be valuable to determine which metric, or perhaps the combination, could help detect early evidence of cerebral metabolic distress.

## Code, Data, and Materials Availability

The datasets generated and/or analyzed during the current study are available from the corresponding author on reasonable request.
